# Urgent need to expand syringe services programs in South Carolina and beyond

**DOI:** 10.1186/s13011-022-00476-0

**Published:** 2022-06-21

**Authors:** Quang Pham, Marc Burrows, Alain Litwin

**Affiliations:** 1 Prisma Health Upstate, 876 West Faris Road, Greenville, SC 29605 USA; 2 Carolina Wellness and Recovery, 103 Clair Drive, Suite C, Piedmont, SC 29673 USA

**Keywords:** Syringe services programs, Harm reduction, Opioid use disorder, Opioid epidemic

## Abstract

Opioid related overdose deaths in the United States claimed over 100,000 thousand lives during the 12-month period ending in April 2021, an increase of 28.5% from the previous period. Syringe services programs (SSPs) are an evidence-based harm reduction strategy that have been shown to be effective in reducing opioid overdose deaths and infectious complications and increasing rates of entry into recovery programs. Ignoring this evidence, South Carolina (SC) and several states have yet to legalize SSPs. In the absence of full legalization, the operation of SSPs in SC faces many barriers. Despite these barriers, Challenges Inc. has been successful in playing a critical role in preventing opioid overdoses through naloxone and fentanyl test strip distribution, reducing infectious complications by providing clean needles, treating individuals with hepatitis C and HIV, and helping patients remain in sustained recovery from opioids. In order for SSPs to function at their full potential to curb the rising tides of opioid overdose deaths and related health complications, policymakers in SC and similar states need to urgently legalize them.

## Background

During the 12-month period ending in April 2021, there were a record high over 100,000 overdose deaths in the United States, an increase of 28.5% from the previous period [1]. Of these deaths, approximately 75,000 were due to opioids. Similar trends were seen with new hepatitis C virus (HCV) infections, which rose by 62.5% from 2015 to 2019 [2]. Fortunately, new HIV infections fell by 11.2% nationally from 2015 to 2019 [3]. Syringe services programs (SSPs) are effective in addressing the opioid epidemic by increasing entry to drug treatment programs fivefold [4], reducing unsafe behaviors of sharing needles by 20–40% [5], and reducing transmission of HCV and HIV by at least 50% each [6, 7]. Additionally, drug treatment programs that use medications for opioid use disorder (MOUD) with either buprenorphine or methadone have been shown to reduce all-cause mortality by 50–67% [8].

According to the North America Syringe Exchange Network (NASEN) there are 370 SSPs in the United States as of 2021. Sixty-nine percent of those SSPs are in urban regions and 63% provided mobile exchange. Services provided range from only HIV and HCV testing to onsite HIV and HCV testing and treatment combined with programs that provide MOUD. As of 2019, thirty-one states and the District of Columbia have laws that explicitly legalize SSPs; nine have laws that reduce barriers to SSPs such as not prohibiting free distribution of syringes; and twelve do not have laws that reduce barriers to SSPs implementation, making SSPs illegal [9]. Because of these restrictions, ten states do not have operating SSPs. Although drug overdose deaths claimed 1785 South Carolinians from February 2020 to February 2021, a 51.8% rise from the previous year [1], South Carolina (SC) does not explicitly authorize SSPs. In SC, there were 17,393 people over the age of 13 years old diagnosed with HIV, and 3817 new cases of HCV identified in 2019 [2, 3].

While SC does not have legislation that authorize the use of SSPs, it also does not have laws that specifically prohibit it. Syringe services programs are not illegal in SC because SC drug paraphernalia law does not specifically mention syringes despite intentionally calling attention to other tools such as bongs and cocaine spoons [10, 11]. However, in the absence of clear legal authorization of SSPs, many organizations are cautious in associating themselves with SSPs due to fear of legal ramifications. The experiences of Challenges Inc., the first SSP of three in SC, help to explain how these legal obscurities translate to the real-life implementation of SSPs. Pending expanded authorization of SSPs, the lessons learned in SC may provide insights into how to effectively provide SSP type services in the states that do not explicitly authorize SSPs.

Founded in 2017 by M.B. (second listed author), Challenges Inc. is a volunteer-run program that has been successful in providing harm reduction services in seven counties in SC. The counties are primarily suburban and rural and have a total population of approximately 1.4 million people [12]. Through support and partnerships with the local community and state agencies, Challenges Inc. has reduced the spread of HIV and HCV through providing over 100,000 sterile syringes, needles and other safe using supplies each year, curbed overdoses by distributing over 15,000 doses of naloxone and fentanyl test strips, with a reported 500+ overdose reversals, connected participants to HIV and HCV treatment, and provided treatment for substance use disorders. All interactions with the legal system have been positive, with police officers wanting to collaborate on providing these services. However, due to the legal confusion around SSPs in SC, funding for Challenges Inc. comes primarily from small foundations and in-kind donations. Here we highlight three success stories of Challenges Inc. to illustrate the effectiveness of an SSP, even in a state without formal legalization of SSPs.

### Success stories of challenges Inc.

An SSP participant is asked about getting point-of-care HIV testing during a regular visit to get clean syringes and naloxone. He tested positive, and the SSP care coordinator provided comfort and education on the new HIV diagnosis and made a warm handoff to the HIV treatment provider. He was started on antiretroviral treatment the next day, and 30 days later his HIV viral load is undetectable.

A second SSP participant who has come in regularly for clean needles, naloxone, fentanyl test strips and other supplies for the last 2 years decided he wants to stop using drugs. He was referred to the co-located clinic which provides MOUD. He received financial assistance to cover his first month of treatment, which included medication and psychosocial therapy, and came in the following week to start treatment. He is now stable on buprenorphine and is actively searching for employment.

A third SSP participant who lives in a transition house was told in the past he may have HCV. He heard about the SSP’s free HCV testing. He came and got tested and is positive for HCV. He was referred to a local primary care provider who provides on-site HCV treatment. He tolerated oral antivirals for hepatitis C well, and he achieved a sustained viral response.

## Discussion

As the success stories with Challenges Inc. illustrate, an SSP can help promote safer drug use behaviors, reduce the onward transmission of infectious diseases including HIV and HCV, increase the rates of entry into MOUD programs, and connect individuals to primary care, even in a state where its existence is not explicitly legal. Syringe services programs have had an even more profound effect in Indiana. In 2015, Austin (Scott County), Indiana experienced the largest concentrated HIV outbreak in the United States [13]. At that time, 5 % of its population became infected with HIV from sharing needles while injecting opioids. The emergency authorization of an SSP at that time helped contained the outbreak [13]. The Centers for Disease Control and Prevention (CDC) has since identified an additional top 220 counties across the US at high risk of HIV and/or HCV outbreaks from drug injection use, with several in states without operating or authorized SSPs [14]. While none of the identified counties are in SC, SC is one of the states deemed “experiencing or at risk of outbreaks” [14].

To prevent further loss of lives from the opioid epidemic and related complications including HIV and HCV, SC and the remaining 18 states without legalized SSPs need to urgently legalize and fund SSPs. While the SC legislature has attempted to authorize SSPs in the past, both attempts have stalled in committees [15, 16]. The ongoing legal ambiguities and perception of SSPs as being illegal have stifled the growth of SSPs. In the case of Challenges Inc., some community partners cannot outwardly support SSPs or allow Challenges Inc. to offer its mobile services on their property due to fear of facing potential legal penalties. Clear laws supporting the use of SSPs would reduce the stigma associated with supportive programs for individuals with substance use disorders and allow rapid expansion. The expansion of these evidence-based programs is urgently needed and would greatly reduce preventable overdoses and drug related mortalities and morbidities.

## Data Availability

Not applicable.
